# Video-assisted thoracoscpic muscle transposition for acute empyema

**DOI:** 10.1186/s13019-015-0332-8

**Published:** 2015-10-08

**Authors:** Hideyuki Maeda, Masato Kanzaki, Takuma Kikkawa, Takamasa Onuki

**Affiliations:** Department of Surgery I, Tokyo Women’s Medical University, 8-1 Kawada-cho, Shinjuku-ku, Tokyo 162-8666 Japan

**Keywords:** Acute empyema, Alveolarpleural fistula, Video-assisted thoracoscopic surgery

## Abstract

Muscle flap transposition is one of the surgical treatment options for empyema with alveolarpleural fistula (APF) or bronchopleural fistula (BPF). This surgical procedure is invasive because it is typically performed by standard thoracotomy. We performed video-assisted thoracoscopic surgery (VATS) debridement, decortication, and obliteration of an empyema cavity using a pedicled latissimus dorsi muscle (LDM) flap harvested through minimal skin incisions for a case of acute empyema with APF. This VATS procedure is effective and less invasive and can be a new option for the thoracoscopic surgical treatment of acute empyema with APF.

## Background

Muscle flap transposition, a surgical procedure used to reduce the empyema cavity, aims for complete cure by closing fistula and filling dead space. The latissimus dorsi muscle (LDM) flap is the largest well-vascularized muscle flap. When the LDM is harvested by traditional thoracotomy, an extensive skin incision is required. We report a novel approach to harvest the LDM flap through minimal skin incisions by video-assisted thoracic surgery (VATS). Debridement, decortication, and pedicled LDM flap transposition for empyema with alveolarpleural fistula (APF) can be accomplished using this less invasive method.

## Case presentation

**Fig. 1:**
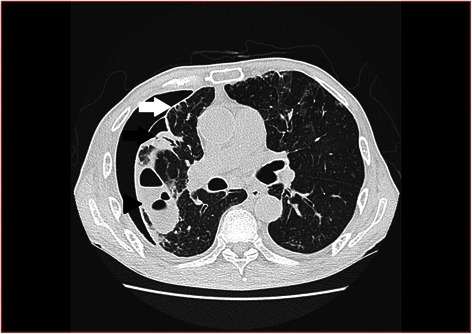
Chest computed tomography shows thickened visceral pleura (white arrow) and an alveolarpleural fistula (APF) (black arrow) at the lateral aspect of the right upper lobe with capsulized interlobar effusion (arrow head)

A 71-year-old man with diabetes mellitus type 2 taking oral hypoglycemic agents was diagnosed with acute empyema, He was admitted to a hospital where chest tube drainage and antibiotics were commenced soon after admission. *Streptococcus pneumonia* was detected in the pleural effusion culture. Although his inflammatory status improved after conservative treatment, an air leak from the chest tube continued and he was subsequently transferred to our hospital. Preoperative chest computed tomography (CT) showed a thickened visceral pleura and an APF covered with a bulla (Fig. 1).

### Surgical technique

**Fig. 2:**
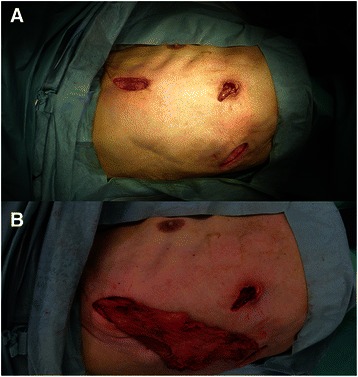
**a** A 5-cm vertical skin incision in front of the anterior edge of the latissimus dorsi muscle (LDM) at the third to fourth rib, a 3-cm skin incision at the sixth and a 5-cm skin incision at the ninth intercostal space. **b** The pedicled LDM flap harvested through small skin incisions

**Fig. 3:**
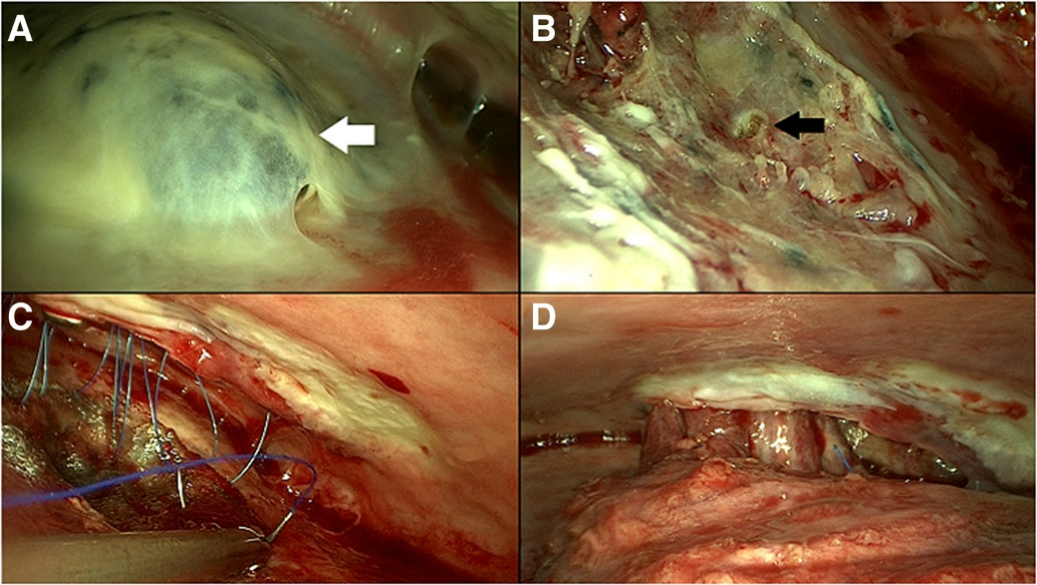
**a** A bulla covering the alveolarpleural fistula (APF) identified at the lateral aspect of the right upper lobe (white arrow). **b** The dissected bulla, exposing the APF (black arrow)**. c** The LDM flap sewn onto the covered area with 9 sutures, and transposed to the empyema cavity through mini-thoracotomy at the third intercostal space. **d** Completed LDM flap transposition

The patient was anesthesized with general anesthesia, intubated with a double-lumen endotracheal tube intubation, and placed in a left lateral decubitus position. A 5-cm vertical skin incision in front of the anterior edge of the LDM at the third to fourth rib, a 3-cm skin incision at sixth rib and a 5-cm skin incision at the ninth intercostal space were made (Fig. 2a). The LDM was accessed, divided from the lower part to the upper part along the muscle fiber, and half of the muscle was eventually harvested (Fig. 2b). A mini-thoracotomy was performed at the third and sixth intercostal spaces, XXS size wound retractors (Alexis® Wound Retractor, Applied Medical, Rancho Santa Margarita, CA, USA) were placed at both places, and a 30°, 10-mm thoracoscope was inserted. A thickened peel was found covering the lung. The clinical phase was fibrinopurulent (StageII) . The APF covered with a bulla was identified beneath the third intercostal space (Fig. 3a). Bubbles from the fistula were recognized while checking for air leaks. The bulla was dissected, and the bed around the fistula was debrided (Fig. 3b). Direct suturing with a 3-0 polybutester (Vascufil™, Covidien, Dublin, Ireland) was used to close the fistula. After confirming that the air leak had disappeared, a polyglycolic acid (PGA) sheet (NEOVEIL®, Gunze, Kyoto, Tokyo) was attached to cover the fistula and the surrounding pleural defect area. Fibrin glue (Bolheal®, Kaketsuken, Kumamoto, Japan) was applied to the covered surface, and VATS debridement and decortication was then performed. The empyema cavity was flushed with warm saline and nine 3-0 polybutester sutures were placed at the edge of the area covered with the PGA sheet to fix the LDM flap under thoracoscopic view. The sutures were placed on the LDM outside the pleural cavity, and tied inside the pleural cavity after the LDM flap was transposed to the empyema cavity through the mini-thoracotomy at the third intercostal space (Fig. 3c, 3d). A 21-Fr chest tube was inserted, and the wounds were closed in layers.

**Fig. 4:**
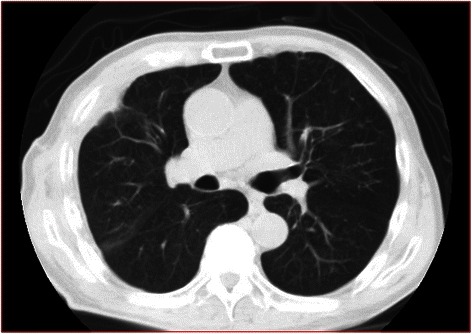
Chest computed tomography image after six months shows right lung expansion and no residual space in the thoracic cavity

There was no postoperative air leak, and postoperative pleural effusion culture was negative. The chest tube was removed on the eighth postoperative day, and the patient was discharged on the tenth postoperative day. He has remained well with no signs of APF recurrence (Fig. 4).

## Discussion

For the management of acute empyema, treatment is primarily based on adequate antibiotic use and proper drainage of the pleural cavity [1, 2]. Previous reports have suggested VATS debridement and decortication as effective and less invasive for patients with acute empyema who are typically in a bad general condition [3, 4]. If acute empyema occurs with APF or BPF, the duration of drainage is prolonged, and some cases progress to chronic empyema. Treatment of empyema with APF or BPF is often complicated. Some cases cannot be treated with a one-stage operation, and a window thoracostomy followed by muscle transposition and thoracoplasty is often needed. Therefore, development of an adequate treatment strategy is required. For our case, we opted for a one-stage VATS debridement, decortications, and obliteration of the empyema cavity using a LDM flap. One-stage operation should be considered for some reasons. First, one-stage operation is beneficial for noncompliant or high-risk patients because the management of such patients with chest drain will be often difficult in outpatients care. In this case, our patient had diabetes mellitus, and he had high risk of recurrence of APF. Second, if initial treatment of at early stage of acute empyema result in failure, or is too late, the nutritional status of the patient will worsen, and it leads to decrease of muscle bulk. It may affect the outcome of the future muscle transposition because small muscle flap cannot fill the space in the pleural cavity.

VATS allowed us to harvest a sufficient amount of the pedicled LDM flap through three small skin incisions. Although we harvested only half of the LDM, in this case it was enough to obliterate the pleural cavity, because the dead space in the pleural cavity shown in the preoperative chest CT was not large space. It is possible to harvest a more extensive muscle flap if necessary. Therefore, this operative method can be an option of treatment for patients with empyema with APF or BPF which has a solid and large space in the pleural cavity requires to be filled with large amount of muscle, or has a large parenchymal leak which cannot be sorted by direct closure or intercostal muscle flap. Transposing muscle flap through mini-thoracotomy can be easily and safely performed with excellent visualization using the thoracoscope. We used a PGA sheet to reinforce the closed APF. Although Tsai *et al.* reported the use of bovine pericardium [5], we prefer bio-absorbable material because infection due to foreign body exposure is a concern. In addition, adhesion between the muscle flap and the APF is expected using a PGA sheet.

## Conclusions

Thoracoscopic surgical treatment for empyema with APF is effective and less invasive than standard thoracotomy. VATS can provide a new treatment option in such cases. Nevertheless, more comparisons with conventional open thoracotomy procedures are needed.

## Consent

Written informed consent was obtained from the patient for publication of this Case report and any accompanying images. A copy of the written consent is available for review by the Editor-in Chief of this journal.

## Abbreviations

APF: Alveolarpleural fistula
BPF: Bronchopleural fistula
VATS: Video-assisted thoracoscopic surgery
LDM: Latissimus dorsi muscle
CT: Computed tomogramphy
PGA: polyglycolic acid
